# Case Report: The phantom gap: a case of congenital pericardial absence revealed by advanced imaging

**DOI:** 10.3389/fcvm.2025.1624625

**Published:** 2025-08-12

**Authors:** Gou Junqi, Yao Fengyou, Liu Chaohui, Lang Mingjian, Jiang Xiaobo

**Affiliations:** Department of Cardiology, Geriatric Diseases Institute of Chengdu/Cancer Prevention and Treatment Institute of Chengdu, Chengdu Fifth People's Hospital (The Second Clinical Medical College, Affiliated Fifth People's Hospital of Chengdu University of Traditional Chinese Medicine), Chengdu, China

**Keywords:** congenital absence of the pericardium, pulmonary embolism, congenital heart disease, pericardium, case report

## Abstract

Congenital absence of the pericardium (CAP) is a rare cardiac anomaly with an estimated prevalence of <1:10,000. CAP results from premature atrophy of the left common cardinal vein during embryogenesis, leading to pericardial defects. In this report, the case of a 39-year-old male with recurrent left-sided chest tightness who was initially misdiagnosed with pulmonary embolism (PE) is presented. Anticoagulation failed to resolve symptoms, prompting advanced imaging and multidisciplinary team review, which confirmed CAP. Conservative management was chosen because of mild symptoms and low herniation risk. This case underscores the diagnostic complexity of CAP and highlights the role of advanced imaging in differentiating CAP from PE. Clinicians should consider CAP in patients with nonspecific cardiac symptoms and imaging findings of cardiac displacement or abnormal mobility.

## Introduction

CAP is a rare congenital anomaly linked to abnormal embryonic pericardial development (1). Approximately 70% of cases involve partial or complete left-sided defects, whereas bilateral or right-sided defects account for <1% of cases (2). Up to 50% of patients are asymptomatic, with CAP often incidentally detected during imaging (3). Symptomatic patients may present with chest discomfort, dyspnoea, or palpitations due to cardiac displacement or coronary compression. Life-threatening complications (e.g., cardiac herniation) are rare but possible (2). Imaging examination is necessary for confirmation of the diagnosis; chest x-ray and echocardiography findings may indicate cardiac displacement, whereas CT angiography or MRI confirms pericardial defects (4). Management ranges from monitoring for asymptomatic patients to surgical repair for high-risk patients.

Here, a case of CAP initially misdiagnosed as PE is presented. We analyse critical diagnostic and management decisions and review embryology, imaging features, and therapeutic advances to improve the clinical recognition of CAP.

## Case report

**Figure 1:**
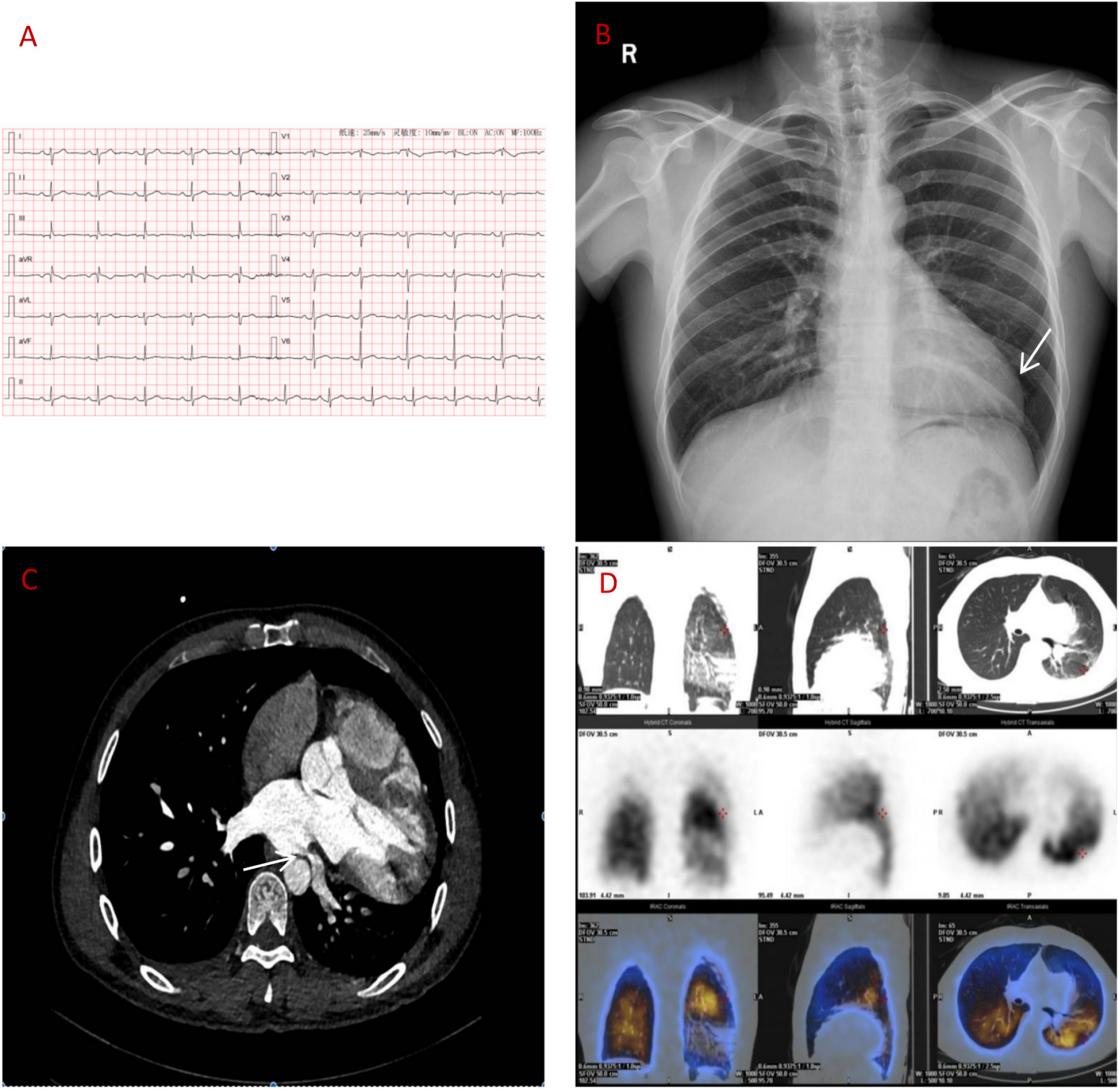
**(A)** ECG: sinus rhythm, incomplete right bundle branch block. **(B)** Chest x-ray: elongated left ventricular contour. **(C)** CT angiography: left atrial compression of the left lower pulmonary vein (over 90%). **(D)** Lung perfusion scan: low perfusion in the left lower lobe.

A 39-year-old man presented with two months of intermittent left-sided chest tightness (5-minute episodes, 2–3 times/month). Prior coronary angiography findings were normal, whereas CT pulmonary angiography findings suggested left lower lobar PE. Right heart catheterization revealed normal pulmonary pressure. Despite therapeutic rivaroxaban (15 mg twice daily) for 4 weeks, his symptoms intermittently persisted. Physical examination revealed a laterally displaced apical impulse (6th intercostal space, midclavicular line). Vital signs and laboratory results (NT-proBNP, CBC, and renal/hepatic function indicators) were unremarkable. Electrocardiography revealed sinus rhythm with incomplete right bundle branch block and T-wave abnormalities (Figure 1A). Echocardiography revealed right ventricular enlargement (52 mm; normal 20–42 mm) and mild mitral regurgitation. Chest x-ray revealed left ventricular elongation (“Snoopy sign”; Figure 1B).

**Figure 2:**
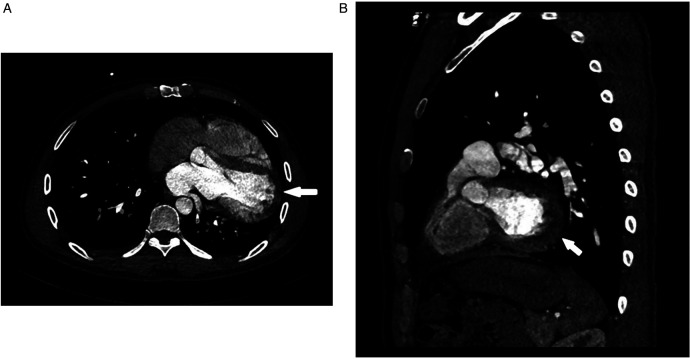
Ct angiography [axial **(A)**, sagittal **(B)** planes]: absence of the left pericardium.

Despite initial imaging suggesting PE, persistent symptoms and negative D-dimer levels contradict typical PE progression. Repeat CT angiography revealed right ventricular enlargement and mild left lower pulmonary vein narrowing (Figure 1C). A lung perfusion scan revealed a small wedge-shaped perfusion defect in the dorsal segment of the left lower lobe, suggesting pulmonary embolism (Figure 1D). Right heart catheterization revealed normal pulmonary artery pressure and vascular resistance. Pulmonary angiography revealed no filling defects or branch truncations in the right pulmonary artery (Pulmonary flow grade 3) or left pulmonary artery. However, delayed contrast flow in the left lower pulmonary artery and venous return (Pulmonary flow grade 2) provided insufficient evidence for pulmonary embolism (5). A multidisciplinary team review revealed leftward cardiac rotation, discontinuity of the left pericardium (Figure 2), and left atrial compression of the lower pulmonary vein, which were consistent with CAP. Given the mild symptoms and low degree of herniation risk, conservative management with follow-up was chosen. At 3-month follow-up (July 2025), the patient remains asymptomatic. This case uniquely highlights pulmonary venous compression due to cardiac displacement-a previously unreported manifestation of CAP-suggesting its clinical variability.

## Discussion

CAP arises from abnormal embryological pericardial development, primarily due to premature regression of the left common cardinal vein (1). The clinical heterogeneity of CAP likely reflects anatomical variations. While 30%–50% of patients remain asymptomatic, common presentations include nonspecific chest pain, dyspnoea, or palpitations. Prior studies emphasize complications such as cardiac displacement, chest pain, coronary compression, and sudden death (6–9); in recent reports aortic dissection (10), ventricular fibrillation (11), and Kommerell diverticulum are described (12). This case uniquely demonstrates left atrial compression of the lower pulmonary vein due to cardiac displacement—a direct consequence of CAP. Although advanced PE management strategies (e.g., catheter-directed thrombolysis) and inflammatory contributors to thrombogenesis are critical in true PE (13), such therapies remain irrelevant here, given the definitive exclusion of thrombotic disease by pulmonary angiography and persistently negative D-dimer. This mechanistic contrast—anatomical compression vs. inflammatory thrombogenesis—underscores CAP's potential to haemodynamically mimic PE while demanding divergent management.

Physical examination, ECG, chest x-ray, and echocardiography are necessary for CAP diagnosis, with confirmation through cardiac CT angiography or MRI. Physical findings typically include leftward displacement of the apical impulse and splitting of the second heart sound, likely owing to cardiac rotation and increased pulmonary artery flow. ECG typically reveals right axis deviation, incomplete or complete right bundle branch block, and poor R-wave progression in precordial leads due to vagal stimulation (2). ST-segment elevation may occur if coronary arteries are compressed by herniated myocardium. Chest x-ray often reveals leftward cardiac rotation, elongated left ventricular contour (“Snoopy sign”), and increased lucency of the aortopulmonary window. However, these findings lack specificity; the absence of tracheal deviation despite cardiac rotation is a distinctive feature (14). Echocardiographic clues include: (1) abnormal acoustic windows, (2) right ventricular enlargement, (3) hypermobile cardiac motion, and (4) abnormal septal movement (15). Right ventricular enlargement may result from increased ventricular compliance due to pericardial absence or cardiac displacement. Cardiac CT angiography or MRI is definitive: CT angiography visualizes pericardial discontinuity and cardiac displacement, whereas MRI dynamically assesses cardiac motion. Combined use increases diagnostic accuracy (4).

There are no standardized treatment guidelines for CAP. Asymptomatic patients are managed conservatively with regular monitoring (e.g., echocardiography or CT angiography every 1–2 years; prompt CT angiography/MRI if new murmurs or worsening symptoms arise) and avoidance of strenuous activity to prevent cardiac herniation (16). Surgical intervention is indicated for patients with the following (17): ① high risk of herniation (defect >3 cm), ② refractory chest pain, or ③ coexisting cardiac anomalies requiring repair. The procedures included pericardioplasty or pericardiectomy.

## Conclusion

This case underscores the importance of integrating advanced imaging and multidisciplinary evaluation to differentiate CAP from PE, particularly when anticoagulation fails. Pulmonary venous compression represents a novel mechanism of CAP-related symptoms, warranting inclusion in diagnostic guidelines.

## Data Availability

The original contributions presented in the study are included in the article/Supplementary Material, further inquiries can be directed to the corresponding author.
